# Hearing Loss in Children: Clinical-Epidemiological Data from Two Different Provinces of the Same Region

**DOI:** 10.3390/audiolres11020017

**Published:** 2021-04-23

**Authors:** Silvia Palma, Andrea Ciorba, Laura Nascimbeni, Mariachiara Pecovela, Laura Negossi, Stefano Pelucchi, Paolo Stagi, Elisabetta Genovese

**Affiliations:** 1 Audiology, Primary Care Unit, 41121 Modena, MO, Italy; 2 ENT & Audiology Unit, Department of Neurosciences, University Hospital of Ferrara, 44124 Cona, FE, Italy; andrea.ciorba@unife.it (A.C.); laura.negossi@unife.it (L.N.); stefano.pelucchi@unife.it (S.P.); 3 Child and Adolescent Mental Health Service AUSL, 41121 Modena, MO, Italy; l.nascimbeni@ausl.mo.it (L.N.); m.pecovela@ausl.mo.it (M.P.); p.stagi@ausl.mo.it (P.S.); 4 Audiology, Department of Diagnostic, Clinical and Public Health Medicine, University of Modena and Reggio Emilia, 41121 Modena, MO, Italy; elisabetta.genovese@unimore.it

**Keywords:** hearing loss, children, newborn hearing screening, early diagnosis

## Abstract

Background: In many countries, neonatal hearing screening programs (NHS) have been available for many years; however, because of the presence of hearing loss at late onset, early hearing detection programs (EHDP) have been implemented. The aim of this study was to evaluate all cases of infantile hearing loss under the care of two different provinces of a regional health service since the introduction of NHS. Methods: Clinical data (the presence of audiological risk factors, age at which children are placed under the care of health service, entity of hearing loss, treatment, and exposure to bilingualism) were retrospectively collected during the period from 1 January 2012 to 31 December 2018, starting from the IT management system used in all of the regional neuropsychiatric services. Results: In total, 124 children were included—116 cases failed the screening, 1 case had an untraceable result, and 7 cases (5.6%) had hearing screening that passed. Most of the children were placed under the care of a neuropsychiatric infantile and adolescence (NPIA) service within the first year of life. The main differences across the two provinces concerned the percentages of audiological risk factors and the number of unilateral hearing loss cases. Conclusion: In order to plan and manage hearing rehabilitation programs for children in the best way, it is very important to know the local clinical-epidemiological features of the population.

## 1. Introduction

Hearing loss is one of the most frequent congenital diseases in children, with a reported prevalence of 1–3:1000 in the healthy baby population, and a major prevalence, with variable results, in the neonatal intensive care unit (NICU) population [1,2]. As it has already been reported, a late diagnosis of hearing loss in children can have consequences on language and cognitive development, depending on the severity of impairment [3]. Therefore, the implementation of newborn hearing screening programs (NHS) and early hearing detection programs (EHDP) is crucial because of the great benefits they have been demonstrated to provide [4].

NHS programs are available, but they do not identify hearing loss at late onset, and some cases may be missed. The Emilia Romagna region approved a document that introduced the newborn hearing screening (NHS) on 1 January 2012, and also guaranteed early intervention programs [5], introducing the hearing disability team (HDT), a multidisciplinary group, which involves the hospital and the territory. HDT is composed of many professionals, such as neuropsychiatrists, audiologists, otorhynolaryngologists, neonatologists, speech therapists, and psychologists, with the task of discussing and implementing a rehabilitative program for each child.

Neuropsychiatrist infantile and adolescent services (NPIA) represent an important resource in the territory, and are provided according to geographical areas and the number of inhabitants. Each NPIA service has a local HDT, and they refer to the regional HDT, which has the role of coordination. The treatments provided by NPIA services are speech therapy, clinical monitoring, and intervention dedicated to school integration.

This study evaluates all cases of infantile hearing loss being treated in NPIA in two different provinces of a regional health service. The main aims were to know how many children participated in an EHDP, as well as the time they were placed in the care of NPIA.

## 2. Patients and Methods

This was a retrospective study, using clinical records. Patients’ data were collected during the period from 1 January 2012 to 31 December 2018, starting from the IT management system used in all of the regional neuropsychiatric services, named ELEA, and searching ICD-10 codes related to hearing loss. In one province, the search was also conducted, using a hospital IT management system named SAP. The data collected included the date and place of birth, gender, presence of audiological risk factors, entity of hearing loss, age of first evaluation by the NPIA service, use of hearing aid/cochlear implant, and exposure to bilingualism.

County 1 had approximately 705,000 inhabitants and it was divided into seven health districts over the territory. County 2 had approximately 343,000 inhabitants and it was divided into three health districts over the territory. For both provinces, each district had its own NPIA service, and families were assigned to the district based on residence. In each county there was a NICU, with five birth facilities in county 1 and two birth facilities in county 2.

Concerning the NHS program, our region adopted a two-stage protocol, differentiating healthy babies from NICU babies. All healthy babies underwent otoacustic emission (OAEs) tests in the birth facilities, and had a re-test within 14 days if there was no clear response. NICU babies were tested by OAEs and by clinical ABR/aABR, because of their increased risk of sensorineural hearing loss and auditory neuropathy [6]. All children, in cases of no clear response, underwent a full audiological evaluation.

Diagnostic hearing testing was conducted by audiologists/otorhynolaryngologists with experience in infantile hearing loss, and normally was completed in the first 3–4 months of age, also depending on the child’s general clinical conditions (very low preterm, Charge syndrome etc.). A full family history was always taken and several other tests could be performed, including tympanometry, visual reinforces audiometry, or play audiometry, depending on the patient’s age and ability to cooperate.

The severity of hearing loss was defined according to the WHO classification, as follows: normal hearing (0–15 db), as well as slight (16–25 db), mild (≥26 to <40 dB), moderate (≥41 to <55 dB), moderate severe (≥56 to <70 dB), severe (≥71 to <90 dB), and profound hearing loss (>90 dB) [7].

The prevalence of hearing loss was calculated for each county by comparing the number of affected subjects with the total number of newborns in the period of the study. Demographic data were obtained from the Regional Health Agency.

## 3. Results

**Figure 1:**
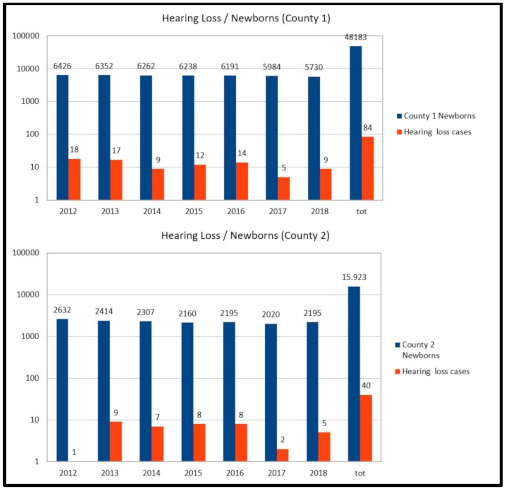
For each county, the total number of newborns per year is indicated (first column), related to the number of new cases of hearing loss treated by territorial services (second column). The prevalence of hearing loss is calculated for the whole period of the study by total number of newborns/number of hearing loss cases, indicated in the last two columns on the right side of the figure.

Globally, 124 children were enrolled in the study. Figure 1 represents the total number of newborns compared with the number of new cases of hearing loss being followed by the NPIA of each county; the analysis was also made per year. The prevalence of hearing impairment cases being treated was 0.17% (84/48183) in county 1 and 0.25% (40/15923) in county 2.

In county 1, 84 children were enrolled in total, of which 75 were born in the region (69 in the province), 7 out of the region, and 2 abroad, and 56 were males and 28 were females.

**Figure 2:**
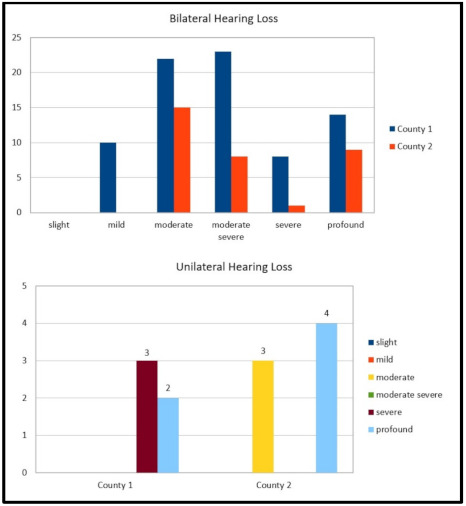
Bilateral and unilateral cases of hearing loss identified in the studied cohorts, according to WHO classification. In very few cases (globally 3) of asymmetrical hearing loss, mainly syndromic pictures, better ear and worse ear did not differ significantly in terms of the hearing threshold. Better ear is intended to indicate the degree of hearing loss.

In total, 79 cases of bilateral hearing loss were identified—14 (17.8%) cases were affected by profound hearing loss, 10 (12.6%) by severe hearing loss, 10 (12.6%) by mild hearing loss (6 were conductive), 22 (27.8%) by moderate hearing loss, and 23 (29.2%) by moderate/severe hearing loss. Five were unilateral cases (about 6%; two profound and three severe; see also Figure 2). Bilateral profound hearing loss cases were due to congenital Cytomegalovirus (cCMV) infection in 3/14 cases (21.4%), to hereditary factors in 3 cases (21.4%), and to prematurity in 1 case (7.2%), with 4 (28.6%) cases not having known factors and 3 (21.4%) cases of syndromes. The prevalence of bilateral hearing impairment cases being treated was 0.16% (79/48,183).

**Figure 3:**
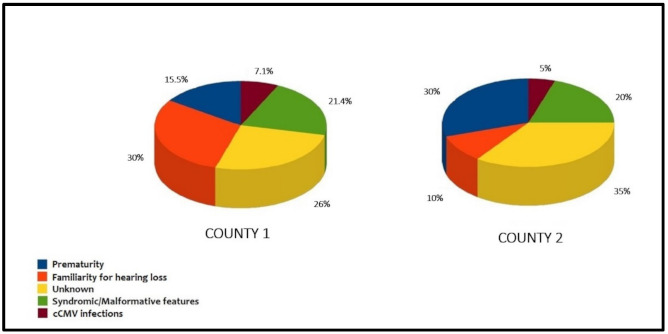
Audiological risk factors in the two examined provinces.

The audiological risk factors identified in the two provinces examined are reported in Figure 3.

Fifty-four cases received hearing aids, 17 recieved cochlear implants, while 13 cases affected by a slight or mild sensorineural or conductive hearing loss (3 of these were unilateral ear malformation) did not receive any hearing rehabilitation. Thirty-nine (46.4%) children were exposed to bilingualism.

**Figure 4:**
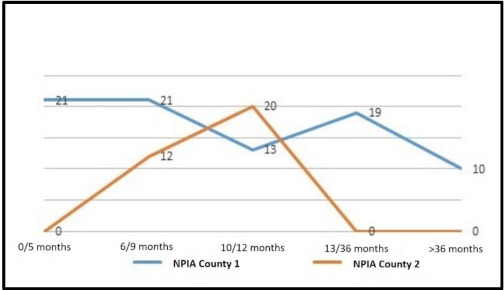
Children placed under the care of neuropsychiatric infantile and adolescence (NPIA) in the examined provinces.

Most of the children (65%) were placed under the care of NPIA within the first year of life, in particular 25% (21 cases) by the fifth month, 25% (21 cases) by the ninth month, and 15% (13 cases) by the first year. Ten cases (12%), affected by mild/moderate hearing loss, were placed under the care of the NPIA service after three years of age (see also Figure 4).

In county 2, 40 children were enrolled in the study. Two children were born out of the province but moved soon after birth, and three children started the intervention program and then moved. Sixteen were males and twenty-four were female. Thirty-three (82.5%) children were affected by bilateral hearing loss. Of these (see also Figure 2), 8 cases were affected by profound hearing loss (24.2%), 1 (3.1%) by a severe hearing loss, 15 (45.5%) by a moderate hearing loss, and 9 (27.2%) by a moderate/severe hearing loss; 7/40 (17.5%) were unilateral (of these, four were profound and three were moderate). Bilateral profound hearing loss was due to syndromes in 2/8 (25%) cases, 3/8 (37.5%) cases were without risk factors, and 3 (37.5%) were very low preterm. The prevalence of bilateral hearing impairment cases being treated was 0.2% (33/15,923).

Twenty- nine cases received hearing aids, seven cases affected by slight or mild hearing loss did not receive any hearing rehabilitation, and eight babies received a cochlear implant. Fourteen (35%) children were exposed to bilingualism.

Twenty babies (50%) were placed under the care of NPIA, mostly by the eighteenth month of life, while the rest of the babies, affected by slight/moderate (or unilateral) hearing loss, were discharged after NPIA service evaluation. In particular, eight children rehabilitated by CI were followed directly by the hospital service, while those with unilateral hearing loss were discharged after the first evaluation (see also Figure 4).

**Figure 5:**
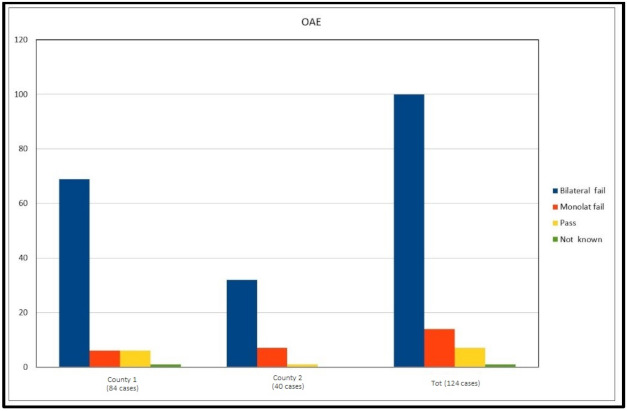
Neonatal hearing screening programs (NHS) results in the two examined provinces.

When analysing the NHS results, in county 1, 77 cases failed the screening (six unilateral fails), in 1 case the result was not traceable, and in 6 cases the screening was passed. In county 2, all except one failed the hearing screening at birth. “Pass” cases at NHS represent 5.6% globally (Figure 5).

In county 1, only one was due to progressive hearing loss with hereditary factors, two were mild with associated disabilities, one was a cCMV infection, and one was moderate due to a cleft palate. In county 2, the only case that was passed on by NHS received a diagnosis of hearing loss by a clinical ABR, performed since the presence of familiarity for hearing impairment.

## 4. Discussion

This study provides a picture of the clinical-epidemiological features of infantile hearing loss treated by territorial services after the introduction of the newborn hearing screening, as well as its implementation through early intervention programs. As evidenced by the data analyzed, almost all children enrolled were identified by the NHS, which achieved optimal coverage in both provinces. Regional health legislation played an important role for achieving these results, underlining the importance of programs receiving strong support from healthcare administrators [8].

Since the analysis of data started by the NPIA IT management system, it has demonstrated that hospital and territorial services have worked concretely in synergy. In particular, children affected by profound bilateral hearing loss have been diagnosed and treated in a timely way, according to the principle that the earlier the intervention, the better the audiological and linguistic outcomes are achieved [9]. The presence of HDT, which is a multidisciplinary and interdepartmental team, constitutes an added value to early intervention programs.

The prevalence of congenital hearing loss had comparable percentages between the two counties. In the literature [1,2,10], most industrialized countries report a prevalence of 1–3:1000 variations observed, and also reflect different criteria in the reporting results [11]. In countries without ongoing NHS programs, prevalence estimates vary between, for example, 19 per 1000 newborns in sub-Saharan Africa and 24 per 1000 in South Asia [12].

In this study, the prevalence of congenital hearing loss was calculated based on the number of cases placed in the care of the NPIA service, and does not include young children who directly receive care in hospital or those who receive NPIA care when they attend primary school. Moreover, a few children with slight or mild hearing loss, identified later, are probably treated in private practice.

The number of patients suffering unilateral hearing loss was found to be significantly different between the provinces, and they were mostly discharged from NPIA after the first evaluation. It is known that, in the past, the impact of unilateral hearing loss was underestimated, and because a child has normal oral language development, the family is likely to assume the absence of implications for child development related to hearing and linguistic abilities.

The results did not reveal particular discrepancies concerning structural abnormalities of the temporal bones and other syndromic clinical pictures, which are amongst the most common causes of permanent congenital sensorineural and mixed hearing loss [12,13]. Similarly, the prevalence of overall congenital Cytomegalovirus infection-related hearing loss, in the examined period, was low, with only a significant difference in the prevalence of cCMV-related profound hearing loss between the two provinces. However, it has been reported that CMV seroprevalence varies between different geographic areas, and can also vary in correlation with ethnicity and socioeconomic status [14].

The main differences across the two provinces was the percentages of prematurity and familiarity for hearing loss, which seemed inversely proportional between the two cohorts. The incidence of hearing loss increased with prematurity [12]; this is a condition that can facilitate the penetration of genetic mutations (mainly recessive types). Instead, the validity of family history as a risk factor for pediatric hearing loss has yet to be fully evaluated [15]. Sutton [16], for example, calculated the prevalence of family history in infants with a congenital hearing loss of 26.2%, very similar to the Modena cohort. The relatively small sample size of the cohorts, at the moment, does not allow for a more in-depth analysis, and a prolonged study of the two groups has been planned.

The presence of bilingualism was slightly different across the examined provinces, also because of the different socioeconomic factors, such as number of inhabitants and the geographic conformation of the two areas. Bilingualism can imply different rehabilitation strategies, mainly when parents are also not skilled in the adoptive language.

Our findings can have implications for the management of available resources in the examined areas and for the organization of the education services specifically oriented to families of those with infantile hearing loss [17]. Indeed, the present study has remarked that it is very important to know the local clinical-epidemiological features of hearing loss in children, in order to plan and manage the economical and human resources in the optimum way. In particular, a well-trained NHS program is crucial for the early diagnosis of hearing loss, such as the audiological follow up of babies with risk factors. Future research should be addressed in order to better understand the possible connections between genetics and prematurity, and to evaluate those cases in which the etiology of hearing loss is still unknown.

The major drawback of this study is the relatively small sample size analyzed.

## 5. Conclusions

The implementation of newborn hearing screening programs represents a very useful tool, both in order to achieve an early diagnosis of hearing loss in children, and in order to establish a timely treatment/rehabilitative protocol. Furthermore, the presence of early hearing detection and intervention programs is crucial in order to treat infantile hearing loss adequately, and this is confirmed in our experience.

## Data Availability

The data presented in this study are available on request from the corresponding author, due to privacy restrictions.

## Abbreviations

The following abbreviations are used in the manuscript:

NICU	Neonatal intensive care unit
NHS	Newborn hearing screening
EHDP	Early Hearing Detection Program
HDT	Hearing disabiliy team
NPIA	Neuropsychiatric infantile adolescence
OE	Otoacustic emissions
ABR	Auditory brainstem response
cCMV	Congenital cytomegalovirus
